# Historical biogeography of *Acer* L. (Sapindaceae): genetic evidence for Out-of-Asia hypothesis with multiple dispersals to North America and Europe

**DOI:** 10.1038/s41598-020-78145-0

**Published:** 2020-12-03

**Authors:** Jian Gao, Pei-Chun Liao, Bing-Hong Huang, Tao Yu, Yu-Yang Zhang, Jun-Qing Li

**Affiliations:** 1 Faculty of Resources and Environment, Baotou Teachers’ College, Inner Mongolia University of Science and Technology, Baotou, China; 2 School of Life Science, National Taiwan Normal University, Taipei, Taiwan; 3 Beijing Key Laboratory for Forest Resources and Ecosystem Processes, Beijing Forestry University, Beijing, China

**Keywords:** Biogeography, Phylogenetics

## Abstract

Biogeography is the study of where, when, and how modern species evolved and diversified. *Acer* L. (maple) is one of the most diverse and widespread genera in the Northern Hemisphere. It comprises 124–156 species in the world, approximately 80% species of *Acer* are native in Asia. The current diversity center of *Acer* is not congruent with the distribution of the oldest fossils of the genus. Therefore, we herein used 84 species and subspecies to reconstruct the phylogeny and investigate the biogeographic history of *Acer* using nuclear ITS and three cpDNA fragments (*psbA*-*trnH* spacer, *rpl16* intron, and *trnL*-*trnF* spacer) with maximum likelihood, maximum parsimony, and Bayesian inference methods. The analyses showed that the current diversity center and the origin center of *Acer* is Asia. Additionally, the North American and Euro-Mediterranean species originated from multiple sources from Asia via the North Atlantic Land Bridge and the Bering Land Bridge, and intercontinental migration has mainly occurred since the Miocene. This study not only provides a novel insight of the origin and dispersal routes of *Acer* but also exemplifies how past climatic changes affect the diversification-rates of Northern Hemisphere forest trees.

## Introduction

The biogeographic studies of widely distributed plants can be performed to provide insight into the broader patterns of the evolutionary history and geographic diversification of the flora of the world^1^. The genus *Acer* L. forms an important part of the Northern Hemisphere forests, and it comprises ~ 129–156 species^2,3^. Most extant species of the genus are native to Asia (about 100 species), whereas others occur in North America, Europe, and North Africa^2–4^. *Acer* fossils dating from the Paleocene to more recent times have been found in the Northern Hemisphere^5–9^. The oldest described fossil of *Acer* is of fossilized fruits from the Paleocene collected in Alaska^10,11^ and is much older than the oldest Asian fossil, which is from the Miocene^8^. As the current biodiversity center of *Acer* and the distribution of the oldest fossil are incongruous^3,4,7^, the two possible explanations of the origin of *Acer* (from North American or Asia; i.e., the hypotheses ‘Out-of-North-America’ or ‘Out-of-Asia’) are evaluated in the present study as an attempt to explain the historical biogeography of *Acer*.

Previous studies on the evolutionary relationships and biogeography of *Acer* have been performed^12–19^. Wolfe^20^ suggested that *Acer* originated in western North America and many lineages spread to eastern Asia during the early Eocene. Hasebe et al.^13^ attributed the current East-Asian and North American distribution of *Acer* to multiple migrations. Renner et al.^19^ also suggested that extant North American species of *Acer* may have derived from their respective Asian clades at different times. However, the evolutionary trajectory of *Acer* is still ambiguous. Moreover, the fact that *Acer* would have experienced climatic oscillations and active orogeny since the Paleocene—for example, the Cenozoic climate cooling^21^, the establishment of the Asian monsoon^22^, and the uplift of the Qinghai-Tibetan Plateau^23^—as well as the Pleistocene climatic oscillations^24^ must be considered.

In addition, because of the large number of *Acer* species worldwide, insufficient sampling is often criticized. Incomplete sampling may lead to bias in molecular dating and ancestral area reconstruction^25,26^. Therefore, an increase in the number of samples of Asian species is necessary to ensure a comprehensive and representative sample size, which in turn would allow the verification of the previous systematic and biogeographic inferences made for the group^3,27^. Because of the possibility of introgression among closely related species of *Acer*^28^, we also used the nuclear ITS to infer the biogeographic history of *Acer* in addition to three chloroplast DNA (cpDNA) fragments (*psbA*-*trnH* spacer, *rpl16* intron, and *trnL*-*trnF* spacer). Based on the results of phylogenetic analyses, we re-evaluate the taxonomic relationships of main sections and series of *Acer*, perform molecular dating with fossil corrections, and predict hypothetical distributions, the original center, and probable dispersal routes of *Acer* species.

## Materials and methods

### Taxon sampling

Eighty-four representative species or subspecies (including varieties) of *Acer* represent the vast majority of sections (except for *Wardiana*) were collected^2^. Among them, there are 11 subspecies (including varieties) derived from one species (different subspecies of the same species). In addition, sixty-seven species are native to Asia, eight to North America and six to Europe, two species are widely distributed in Europe and Asia, and one species is distributed in North America and Asia (Table S1).

Species identification and classification followed the *Maples of the World*^2^. *Aesculus* L. and *Dipteronia* Oliv. were chosen as outgroups for phylogenetic analyses^29,30^. Detailed sampling information is listed in Table S1.

### Molecular methods

Total genomic DNA was extracted from silica-dried leaves using a modified CTAB method^31^. The primer pairs used for PCR amplification and sequencing are listed in Table S2. PCR amplification was conducted in a LABNET MultiGene 96-well gradient thermal cycler, with a reaction volume of 20 μL containing 10–30 ng of DNA template, 50 mM Tris–HCl, 1.5 mM MgCl_2_, 1 mM dNTPs, 0.3 μmol/L of each primer pair, and 0.2 U of Taq DNA polymerase (Bernardo Biotech Co., Taiwan). PCR cycles were as follows: 3 min at 94 °C, 30 cycles of 30 s at 94 °C, 30 s at 50–58 °C, and 45–80 s at 72 °C, with a final elongation for 10 min at 72 °C. The PCR products were separated by agarose gel electrophoresis and purified with a Gel Band Purification Kit (TIANGEN Biotech Co., Beijing, China). The purified PCR products were directly sequenced with the PCR primers using the ABI 3730XL DNA analyzer (Applied Biosystems, USA). The sequences are deposited in GenBank under accession numbers KU500429–KU500554 and KU522488–KU522550.

### Sequence alignment and phylogenetic analyses

Sequence contigs were assembled and edited using SeqMan implemented in DNAStar version 7.0. Sequence alignments were conducted using BioEdit version 7.1.11^32^ and manually refined. The 5ʹ and 3ʹ ends of sequences were trimmed to equal alignment lengths for subsequent phylogenetic analyses. The phylogenetic tree with nuclear ITS, three cpDNA fragments and a combination of four DNA fragments were reconstructed separately. A partition homogeneity test^33^ was performed to test the homogeneous evolutionary rates of four DNA fragments for ensuring the rationality of combining them as one locus. Combining sequences from different sources is a common practice in the phylogenetic analyses^26,34,35^.

Phylogenetic relationships were reconstructed under maximum parsimony (MP), maximum likelihood (ML), and Bayesian inference (BI) using PAUP^*^4.0b10^36^, GARLI v0.951^37^, and MrBayes 3.2^38^, respectively. For the whole dataset, the best-fitting substitution model for the ML and BI analyses was inferred using Modeltest 3.7^39^. In the MP analysis, all character states were treated as unordered and equally weighted, and a heuristic search was performed with 1000 replicates of random addition of sequences, tree-bisection-reconnection (TBR) branch-swapping, and MULTREES on. Bootstrap analysis was conducted with 1,000 replicates using the same heuristic search settings as described above. The ML analysis was initiated from a BIONJ tree, with support values for the nodes estimated by 1000 bootstrap replicates. In the Bayesian analyses, two independent Markov Chain Monte Carlo (MCMC) runs were initiated, each consisting of one cold and three heated MCMC chains that were run for 5,000,000 generations and sampled every 1000 generations. The first 1000 trees were discarded as burn-in to ensure that the chains had become stationary. The clades division in figures is mainly based on the clustering results of phylogenetic trees and the morphological classification of *Acer*.

### Molecular clock dating

Two fossil calibrations were used to constrain (1) the divergence time of *Acer* and *Dipteronia* and (2) the crown age of *Acer*. Although *Acer* has a rich fossil record, most fossils are difficult to identify as close relatives of extant species because of the limited morphological differentiation among species. Because of a lack of radiometric dating for fossils, Renner et al.^19^ was followed to adjust the ages of the fossils to the midpoint of the currently assigned range for each respective geological Epoch or Stage. Therefore, the divergence time of *Acer* and *Dipteronia* and the crown age of *Acer* were calibrated to 62 million years ago (Mya) and 53 Mya with a shape parameter of 4.0 and 1.0, respectively. Divergence times were estimated using BEAST v.1.7^40^, with a relaxed uncorrelated normal clock model of speciation. The MCMC analysis was run for 1,000,000,000 generations. Trees were sampled every 50,000 generations, with the first 4000 trees treated as burn-in.

### Ancestral area reconstruction

BioGeoBEARS^41^ as implemented in R was used to infer the biogeographical history of *Acer*. This program allows estimation of ancestral ranges under different models such as DEC^42,43^, a likelihood interpretation of DIVA^44^ (DIVALIKE) or a likelihood interpretation of BayArea^45^ (BAYAREALIKE). Additionally, it implements a parameter describing founder-event speciation (+J, post-colonization cladogenesis) likely important in oceanic settings^46^ and allows the comparison of different models in a statistical framework. The analyses were carried out based on the BEAST Maximum Clade Credibility (MCC) tree with outgroups removed. The study species were mapped in four study areas: (A) Asia, (B) Euro-Mediterranean, (C) western North America, and (D) eastern North America. A random sample of 16,000 post-burn-in trees generated from the BEAST analysis for ancestral area reconstruction was used. The analysis was run on the chronogram obtained under the fossil-calibrated relaxed clock. Biogeographical data were coded according to the distribution of extant taxa included in this study.

### Temporal-based diversification-rate-shift analyses

The temporal-based analysis that accumulates speciation events (lineages) through time was conducted for inferring the emergence time of species via the lineage-through-time (LTT) analysis. The LTT analysis was performed in R package (APE)^47^. Effective lineage sizes through time were also estimated using the reversible jump Markov Chain Monte Carlo (rjMCMC)^48^ by the function mcmc.popsize of APE with one-million-steps Markov chain simulations, and discarding the first 1,000 steps as burn-in, with a setting thinning factor = 1000 for both whole tree topologies.

## Result

### Sequence characteristics

Sequences with 533, 609, 995, and 1046 base pairs (bp) of ITS, *psbA*-*trnH* spacer, *rpl16* intron, and *trnL*-*trnF* spacer were obtained, respectively. In the 3183 bp of combined sequences, 594 variable sites with 396 parsimonious informative sites were obtained when *Aesculus* and *Dipteronia* were used as outgroups.

### Phylogenetic relationships

**Figure 1:**
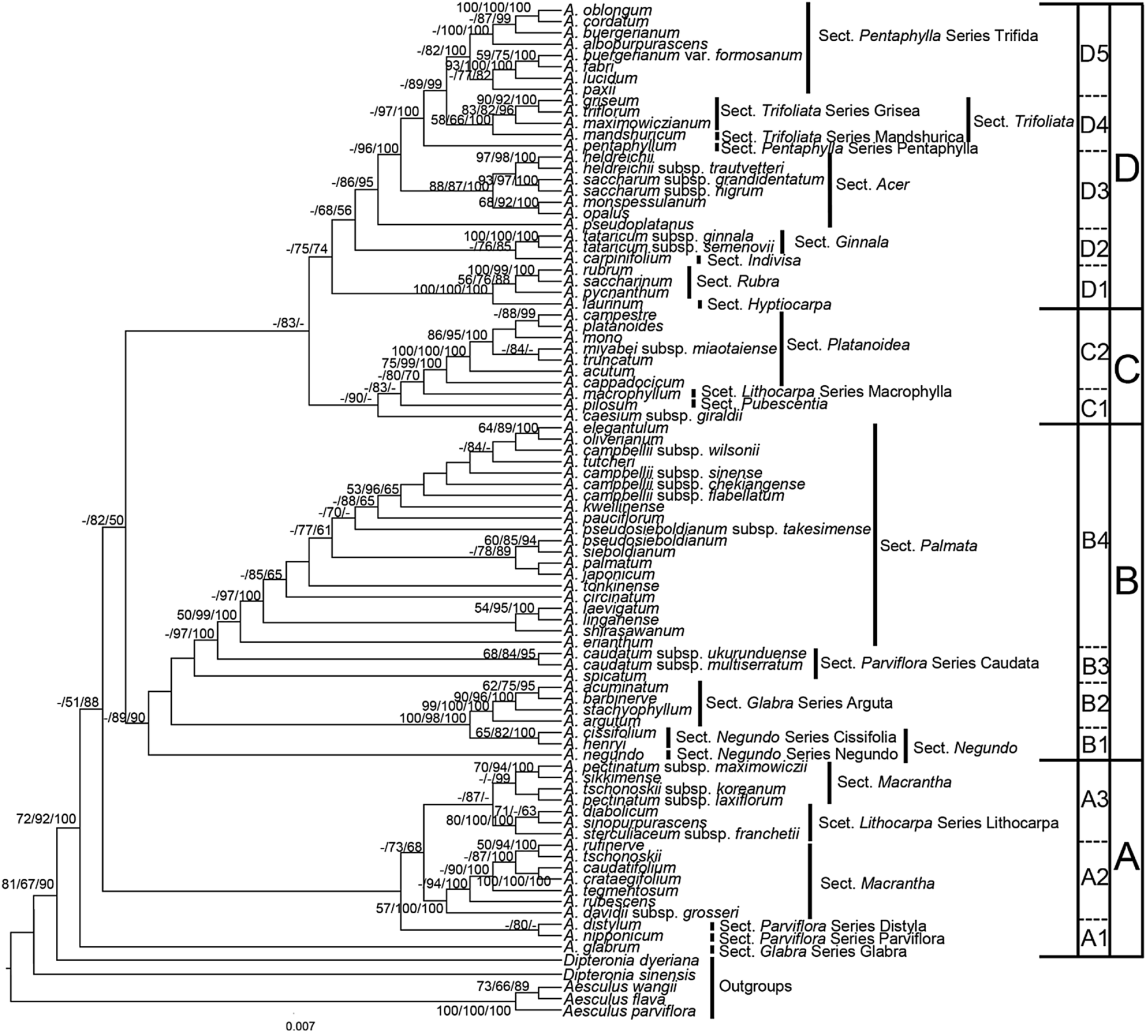
The best maximum likelihood tree combined nuclear ITS and three cpDNA fragments (*psbA*-*trnH*, *rpl16* and *trnL*-*trnF*). The tree is rooted using *Aesculus* and *Dipteronia* as outgroups. Bootstrap values of MP, ML above 50% and posterior support value of BI above 0.5 are shown successively. The diagram was generated by Microsoft PowerPoint 2019 (https://www.microsoft.com/zh-cn/microsoft-365/powerpoint).

We reconstructed the phylogenetic trees with ITS (Fig. S1), three cpDNA fragments (Fig. S2) and a combination of four DNA fragments. These trees have consistent topologies with only slight differences within some subclades, which is probably due to few informative sites if reconstructing the phylogenetic tree by single genes only. In addition, non-significant incongruence was found in substitution rates among four DNA fragments by the partition homogeneity test (*P* = 0.08). Therefore, we showed the tree reconstructed by concatenated DNA fragments in Fig. 1. We used three approaches, ML, MP, and BI, to reconstruct the phylogenetic trees of four DNA fragments (Figs. 1, S3, S4). These approaches resulted in roughly congruent phylogenetic topologies except for slight differences in the grouping of certain tips (the best model for the ML and BI analyses was GTR+G+I). The ML tree with more polytomies represented a more conservative tree topology than the other approaches, and it is therefore the main object of this discussion (Fig. 1).

The result strongly supports the monophyly of *Acer* (72/92/100) with four major clades, namely clades A, B, C, and D (Fig. 1). In the A2 and A3 branches, the series *Lithocarpa* of section *Lithocarpa* formed a clade (80/100/100), whereas the other species belonged to section *Macrantha* (Fig. 1). Series *Cissifolia* of section *Negundo* (65/82/100) and series *Arguta* of section *Glabra* (99/100/100) formed monophyletic groups in branches B1 and B2, respectively. Branch B4 was mainly composed of the series *Palmata* and series *Sinensia* species of section *Palmata*. Branch C2 was mostly formed by section *Platanoidea* species with a relatively high support value (75/99/100). Branch D1 consisted of section *Rubra* (including several North American species, such as *A. rubrum* and *A. saccharinum*) and section *Hyptiocarpa* (100/100/100). Branch D2 was composed of section *Ginnala* (100/100/100) except *A. carpinifolium*. Branch D3 was composed of section *Acer* (including several European species, such as *A. heldreichii*, *A. monspessulanum*, and *A. opalus*) (88/87/100) except *A. pseudoplatanus*. Branch D4 was formed by section *Trifoliata* (58/66/100) except *A. pentaphyllum*. Branch D5 was mainly composed of the series *Trifida* of section *Pentaphylla* and series *Penninervia* of section *Palmata* (Fig. 1).

### Divergence time estimation

**Figure 2:**
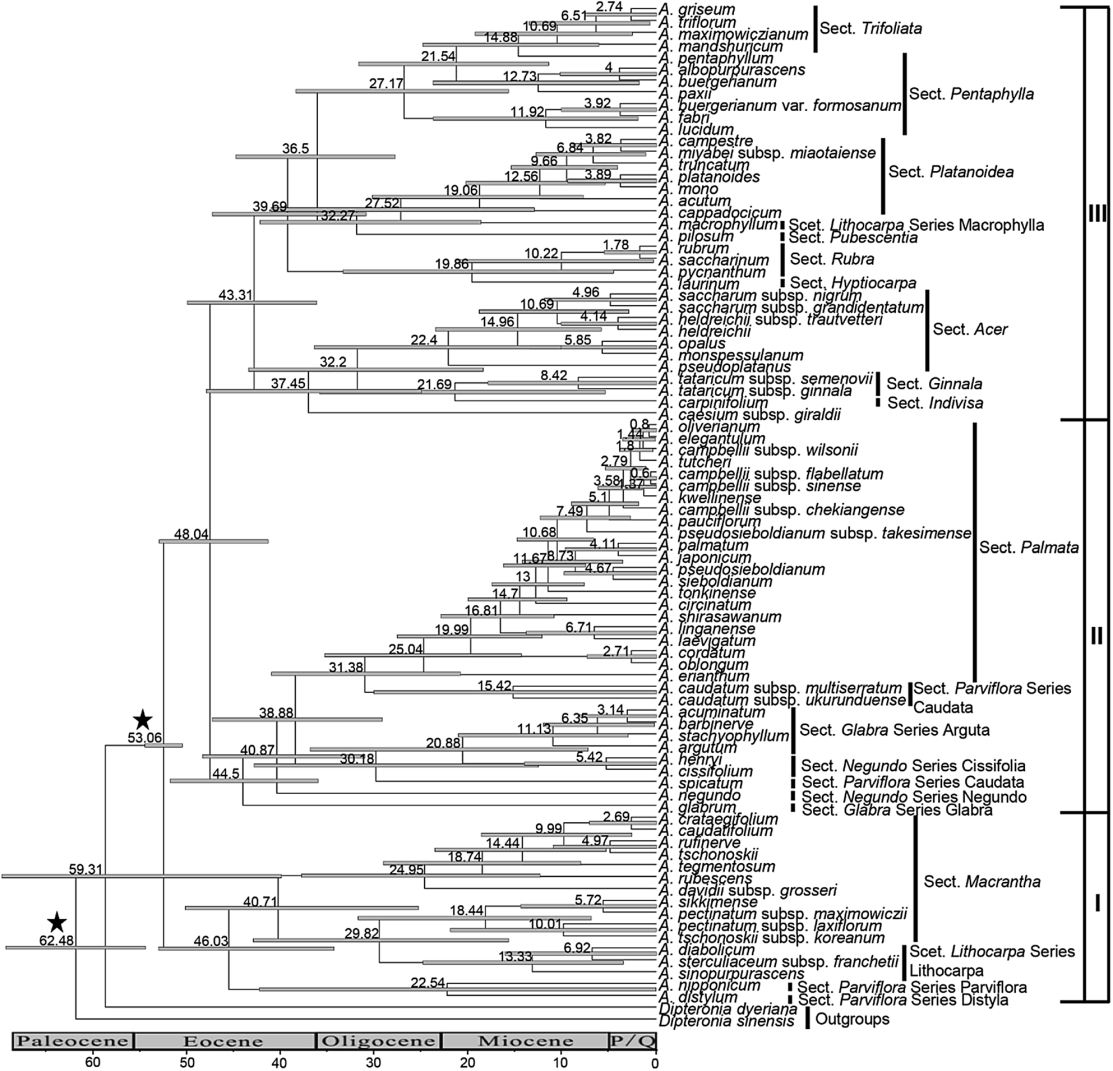
Chronogram for *Acer* reconstructed based on the combined data matrix (nuclear ribosomal ITS, plastid *psbA*-*trnH* spacer, *rpl16* intron, and *trnL*-*trnF* spacer). Stars indicate the calibration points inferred from fossil records. Numbers on nodes indicate time to the most recent common ancestor (MRCA). Each numbered node bar represents the minimum and maximum ranges of dates for MRCA summarized from both Low and High calibration. The diagram was generated by Microsoft PowerPoint 2019 (https://www.microsoft.com/zh-cn/microsoft-365/powerpoint).

Three main clades (I, II, and III) were inferred by the Bayesian chronogram BEAST (Fig. 2). The results of this analysis were similar to those of the ML, MP, and BI analyses with a slight difference in the placement of some statistically unsupported nodes (Figs. 1, S3, S4). The relaxed molecular clock analysis with two calibration points indicated that the estimated crown ages of clades I, II, and III are 46.03 Mya (95% Highest Posterior Density interval (HPD): 34.71–53.59), 44.50 Mya (95% HPD: 36.40–52.35), and 43.31 Mya (95% HPD: 36.54–50.51), respectively, and roughly in the middle Eocene. Moreover, it indicated that most of the sections and series of *Acer* originated from the end of the Oligocene to the early Miocene (e.g., sections *Palmata* and *Macrantha*) (Fig. 2).

**Figure 3:**
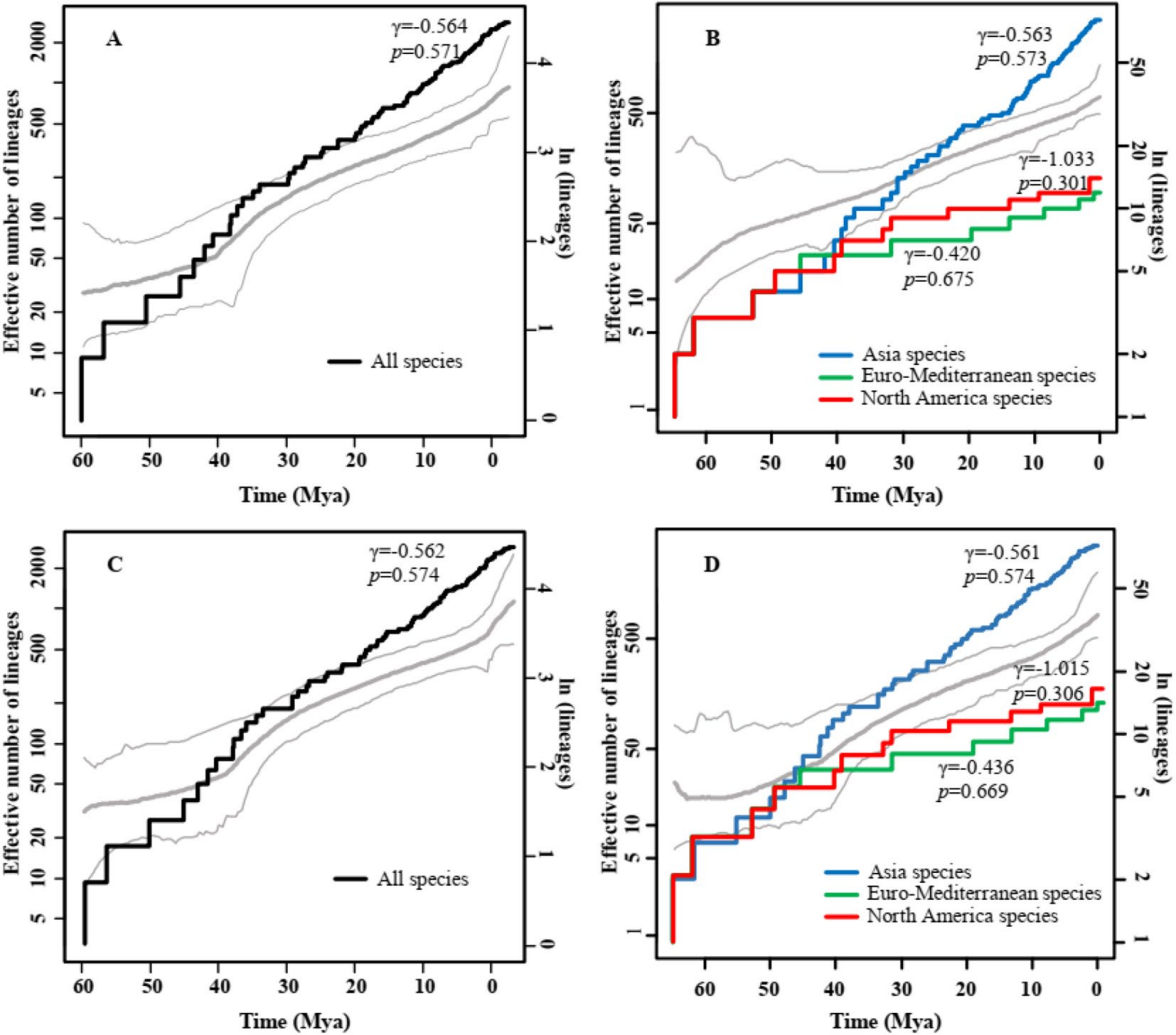
Temporal analysis of diversification rates. The lineage-through-time (LTT) of *Acer* species and reversible jump Markov chain Monte Carlo (rjMCMC) simulation of *Acer* (gray curves: bold line indicates mode value, and thin lines indicate 95% confidence intervals; left y-axis). (**A**) All species (dark curve, left y-axis). (**B**) Species from different regions (color curve, left y-axis). (**C**) All species except 11 subspecies (including varieties) which derived from one species (different subspecies of the same species) (dark curve, left y-axis). (**D**) Species (except 11 subspecies) from different regions (color curve, left y-axis). The diagrams of (**A**–**D**) were generated by Microsoft PowerPoint 2019 (https://www.microsoft.com/zh-cn/microsoft-365/powerpoint).

The LTT plot showed a trend of exponential lineage growth in the width of the phylogenetic tree on a logarithmic scale suggesting that the diversity rate of all *Acer* species remained stable (Fig. 3A,C). Figure 3B,D shows the diversity rate diverging approximately 30 Mya among different distributions, with a notable decline in North America and Europe.

### Biogeographic analyses

**Figure 4:**
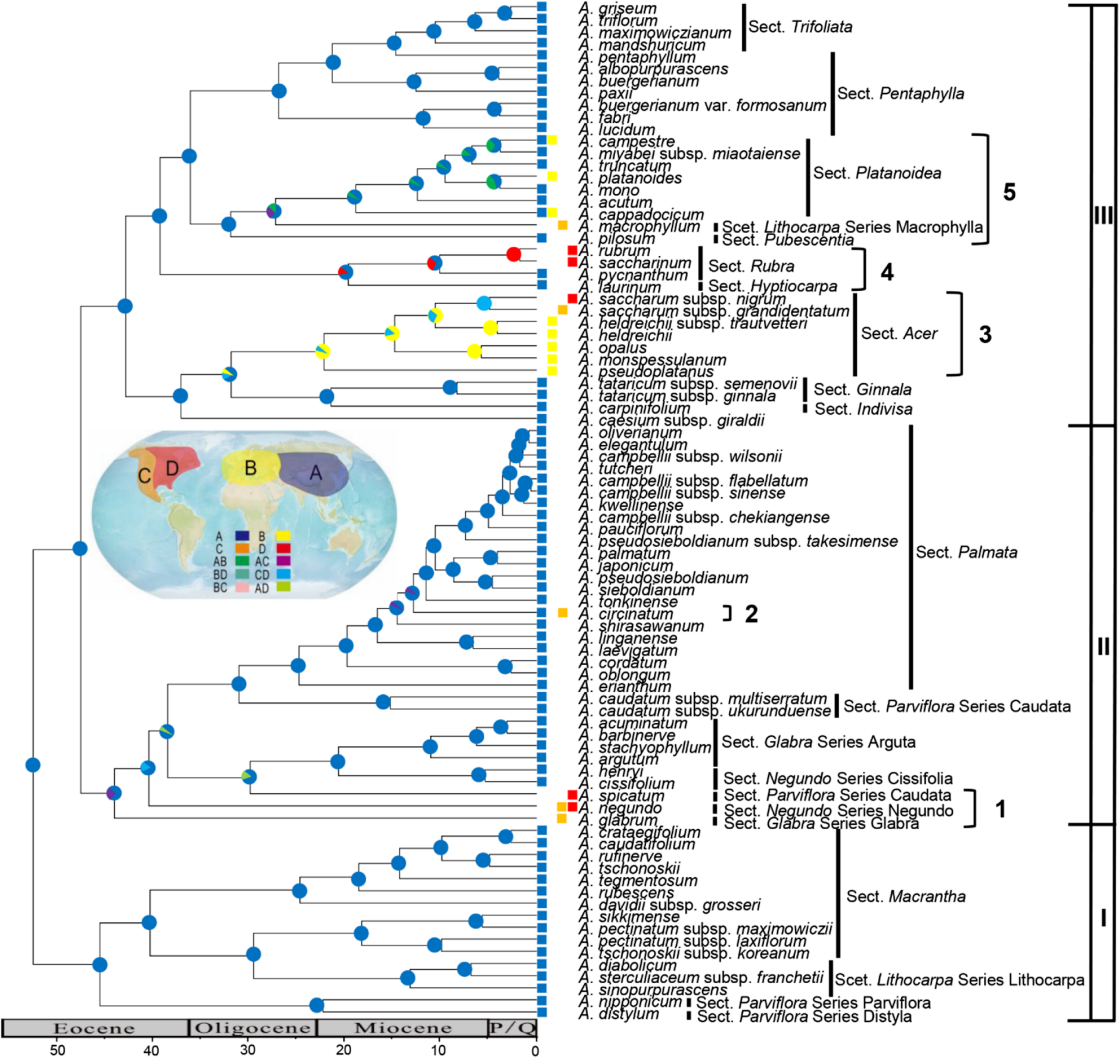
Ancestral area reconstruction for the genus *Acer* based on the combined data matrix (nuclear ribosomal ITS, plastid *psbA*-*trnH* spacer, *rpl16* intron, and *trnL*-*trnF* spacer). The one- and two-letter codes in the inset represent different geographic ranges. The map shows the geographical extents of the four areas ((**A**) Asia, (**B**) Euro-Mediterranean, (**C**) western North America, (**D**) eastern North America) considered in the analysis. The pie charts on the nodes show the most likely ancestral areas (correspond to the colors in the inset) as reconstructed by BioGeoBEARS. Current distributions are indicated before the species names. Branches 1–5 represent five migration events of *Acer*. The diagram was generated by Microsoft PowerPoint 2019 (https://www.microsoft.com/zh-cn/microsoft-365/powerpoint).

The best biogeographic model for the genus *Acer* as evaluated by BioGeoBears was DEC+J (AIC = 147.67, ln*L* = − 70.83) (Table 1). This analysis showed that the most probable ancestral area of *Acer* is Asia (region A), whereas the extant Euro-Mediterranean and North American species were distributed in different branches (Fig. 4). Moreover, the analysis showed that at least five independent dispersal events from Asia to North America in clades I–III are needed to explain the present differences in the Asia-North America distribution pattern (Fig. 4). The timing of most dispersal events was mainly in Miocene and a few in the Eocene (Fig. 4). In clades II, the effective number of lineages after 40 Mya in European and American species has declined, indicating that their number of species has not increased.

**Table 1:** Models and parameters in Fig. 4 conducted using BioGeoBEARS.

Model	*n*	*d*	*e*	*j*	In*L*	AIC
DEC	2	0.0031	0.0000	0.0000	− 80.21	164.42
**DEC+J**	**3**	**0.0009**	**0.0000**	**0.0208**	− **71.17**	**148.34**
DIVALIKE	3	0.0016	0.0000	0.0164	− 72.21	150.44
DIVALIKE+J	3	0.0016	0.0000	0.0164	− 72.21	150.44
BAYAREALIKE	3	0.0008	0.0000	0.0245	− 77.51	161.02
BAYAREALIKE+J	3	0.0016	0.0000	0.0164	− 72.22	150.44

Number of parameters (*n*), Dispersal (*d*), Extinction (*e*), Founder (*j*), values of Log-Likelihood (ln*L*) and Akaike Information Criterion (AIC) scores from each model implemented.

The best model is highlighted in bold.

## Discussion

### Phylogenetic relationships

Similar to the results of previous studies, the present results support the monophyly of *Acer*^16–19^ (Fig. 1). Based on leaf and fruit characteristics, Ogata^49^ defined three series in section *Macrantha*. However, the phylogenetic inference herein obtained in section *Macrantha* is divided into two parts in clade A (Fig. 1) does not support such taxonomic treatment; it is however congruent with the taxonomic proposal of Zhang et al.^50^. Series *Palmata*, *Sinensia*, and *Penninervia* of section *Palmata* in the B4 and D5 branches do not form a monophyletic group. Reciprocal paraphyly may reflect the frequent interbreeding among *Acer* species^51^ or the previous misclassification. Renner et al.^19^ found a phylogenetic admixture between species of sections *Rubra* and *Hyptiocarpa* based on cpDNA only. However, both ITS and cpDNA used in the present study suggest a monophyly of section *Rubra* separate from section *Hyptiocarpa* (Fig. 1). These incongruent phylogenetic inferences may be a result of incomplete lineage sorting caused by ancestral interbreeding. The Euro-Mediterranean and North America species did not form the monophyletic group (Fig. 1), which is consistent with the results of Renner et al.^19^. This may indicate the multiple origins of the Euro-Mediterranean and North America species.

### Diversification rate of *Acer*

The diversification rates of *Acer* in the Euro-Mediterranean and North American regions began to decline approximately 30 Mya (mid-Oligocene) (Fig. 3B,D). This may have been caused by the limitation of migration in the Euro-Mediterranean and North American regions because of global climate cooling with the onset of permanent Antarctic ice sheets^52^. Compared to Asia, the Euro-Mediterranean and North America regions have a low number of species, which may also contribute to the decline of species diversification. In addition, most areas of Euro-Mediterranean and North America were covered by ice sheets during glacial periods; this may have led to a higher extinction rate among species in these areas than in those in Asia, which did not experience large areas of glacier cover^52^.

### Biogeographic patterns of *Acer*

The phylogenetic analyses showed that the most probable ancestral area of *Acer* was Asia (Fig. 4), even though the oldest fossil of *Acer* dating to the Tertiary period was discovered in North America^10,11^. Asia was found to be the modern diversity center of *Acer* with many primitive species^53^. The entire area of the north–south trending river valleys in southwest China acted as an important migration corridor, offering protection during cycles of climate oscillation^54^. Therefore, the origin center of *Acer* is speculated to be in Asia, from where the genus was later dispersed to Europe and North America (Fig. 4). In clade II, the North American species is most close to the root of clade II, makes it possible that the North American species was the origin of species of clade II. However, Asian origin still has the highest probability in the assessment of the ancestral area of clade II because the geographic distributions of branches other than clade II are also inferred to be in Asia. Although we cannot completely rule out the hypothesis of the Extinction of North American ancestors, from the geographic distribution and phylogenetic positions of extant species, the Out-of-Asia is the most parsimonious hypothesis of the biogeographic pattern of *Acer*. In addition, the decline in the effective number of lineages of European and American species after 40 Mya may be due to the fact that the extinction rate was similar to the speciation rate, and the immigration rate (from Asia) of the European and American species has declined after 40 Mya.

Modern genera with global distributions have been hypothesized to have originated in various areas and attained their present distribution via multiple pathways. At least three possible dispersal routes have been proposed to explain the intercontinental distribution of *Acer*: migration across the NALB^55^, migration across the BLB^56^, and long-distance dispersal^1,56,57^.

Our results allow us to hypothesize that, in the middle to late Eocene, *Acer* species migrated from Asia to North America forming *A*. *glabrum*, *A*. *negundo*, and *A*. *spicatum* in branch 1 (Fig. 4). The BLB connected East Asia and North America from the late Eocene to the middle Oligocene, providing a corridor for species migration^1^. In branches 2–5, migration events are hypothesized to have occurred mainly in the Miocene (Fig. 4). *A*. *circinatum* (branch 2), *A*. *rubrum* (branch 4), *A*. *saccharinum* (branch 4), and *A*. *macrophyllum* (branch 5), currently distributed in North America, probably migrated from Asia via the BLB. The BLB provided a route for temperate migration between Asia and North America until ca. 3.5 Mya^1,58^. Many species have been suggested to have migrated using the BLB, such as *Penthorum* L. (Penthoraceae)^59^, *Circaea* L. (Onagraceae)^60^, and *Saxifraga rivularis* L. (Saxifragaceae)^61^. Therefore, migration events using the BLB seem to be the most reasonable explanation for the intercontinental distribution of taxa in Asia and North America. However, the Aleutian land bridge, south of the BLB in the Tertiary, has also been proposed as an explanation for these migration routed^1^.

The NALB between Eurasia and eastern North America is another possible migration route (Fig. 4). *Acer* species from branch 3 are hypothesized to have migrated to Europe and formed four endemic species (*A*. *pseudoplatanus*, *A*. *monspessulanum*, *A*. *opalus,* and *A*. *heldreichii*). Then, some species may have migrated from Europe to North America and formed *A*. *saccharum* subsp. *nigrum* and *A*. *saccharum* subsp. *grandidentatum* (Fig. 4). The NALB was the shortest route connecting Europe and North America. Tiffney^55,56^ suggested that plant migration via the NALB was possible during the Paleocene and Eocene. Even in the Miocene, the islands could have served as “stepping stones” for the migration of deciduous taxa from Europe to North America^1^. In summary, we propose that *Acer* originated in Asia, dispersed to Europe, and spread to North America through different routes (namely, NALB and BLB).

During the Oligocene–Miocene transition, the global climate warming trend reduced the extent of the Antarctic ice sheets^62^; this warm phase peaked in the mid-Miocene Climatic Optimum (17–15 Mya)^63,64^. Donoghue and Smith^65^ summarized the discontinuous distribution pattern of 66 plant groups and concluded that most temperate forest plants originated and diversified in Asia. These plants spread out from Asia several times since 30 Mya, and Miocene was an active periods of species diffusion. In addition, the considerable increase of insect diversity in the Miocene may accelerate the diversification rate of plant species^66^. Therefore, it is reasonable to conclude that climate change during the Miocene may have been a major driving force for the migration of *Acer*.

## Conclusion

In this study, we not only reduced the taxonomic problem surrounding *Acer* by reconstructing a phylogeny but also raised the hypothesis of Asian origin and multiple long-distance dispersal routes for the species in this genus. Regarding large-scale biogeographic patterns, the case of this genus shows the impact of climate change on dispersal and speciation, which in turn affects the diversification rate among continents. Asia, the origin center and the region less covered by ice sheets during glacial periods, became a hotbed for the growth of *Acer* and its speciation. The diverse landforms, such as Hengduan Mountain, are also beneficial to species diversification as they provide rich niches. In conclusion, this study provides an outstanding example for the discontinuous distribution in the north temperate zone.

## Supplementary information

Supplementary Information.

## Data Availability

All data sets are provided in the Supplementary Information and deposited in GenBank.
