# Supplementary material for: Historical biogeography of Acer L. (Sapindaceae): genetic evidence for Out-of-Asia hypothesis with multiple dispersals to North America and Europe
Source: Sci Rep. 2020 Dec 3;10:21178. doi: 10.1038/s41598-020-78145-0 (PMC7712834; doi:10.1038/s41598-020-78145-0)
Supplement: Supplementary file 1 — Supplementary Information. [file 41598_2020_78145_MOESM1_ESM.docx]

Supplementary Material

**Historical Biogeography of *Acer* L. (Sapindaceae): Genetic Evidence for Out-of-Asia Hypothesis with Multiple Dispersals to North America and Europe**

Jian Gao^1^, Pei-Chun Liao^2,*^, Bing-Hong Huang^2^, Tao Yu^3^, Yu-Yang Zhang^3^, Jun-Qing Li^3,*^

^1^Faculty of Resources and Environment, Baotou Teachers' College, Inner Mongolia University of Science and Technology, Baotou, China

^2^School of Life Science, National Taiwan Normal University, Taipei, Taiwan

^3^Beijing Key Laboratory for Forest Resources and Ecosystem Processes, Beijing Forestry University, Beijing, China

***Correspondence:**

Dr. Pei-Chun Liao

pcliao@ntnu.edu.tw

Dr. Jun-Qing Li

[lijq@bjfu.edu.cn](mailto:lijq@bjfu.edu.cn)


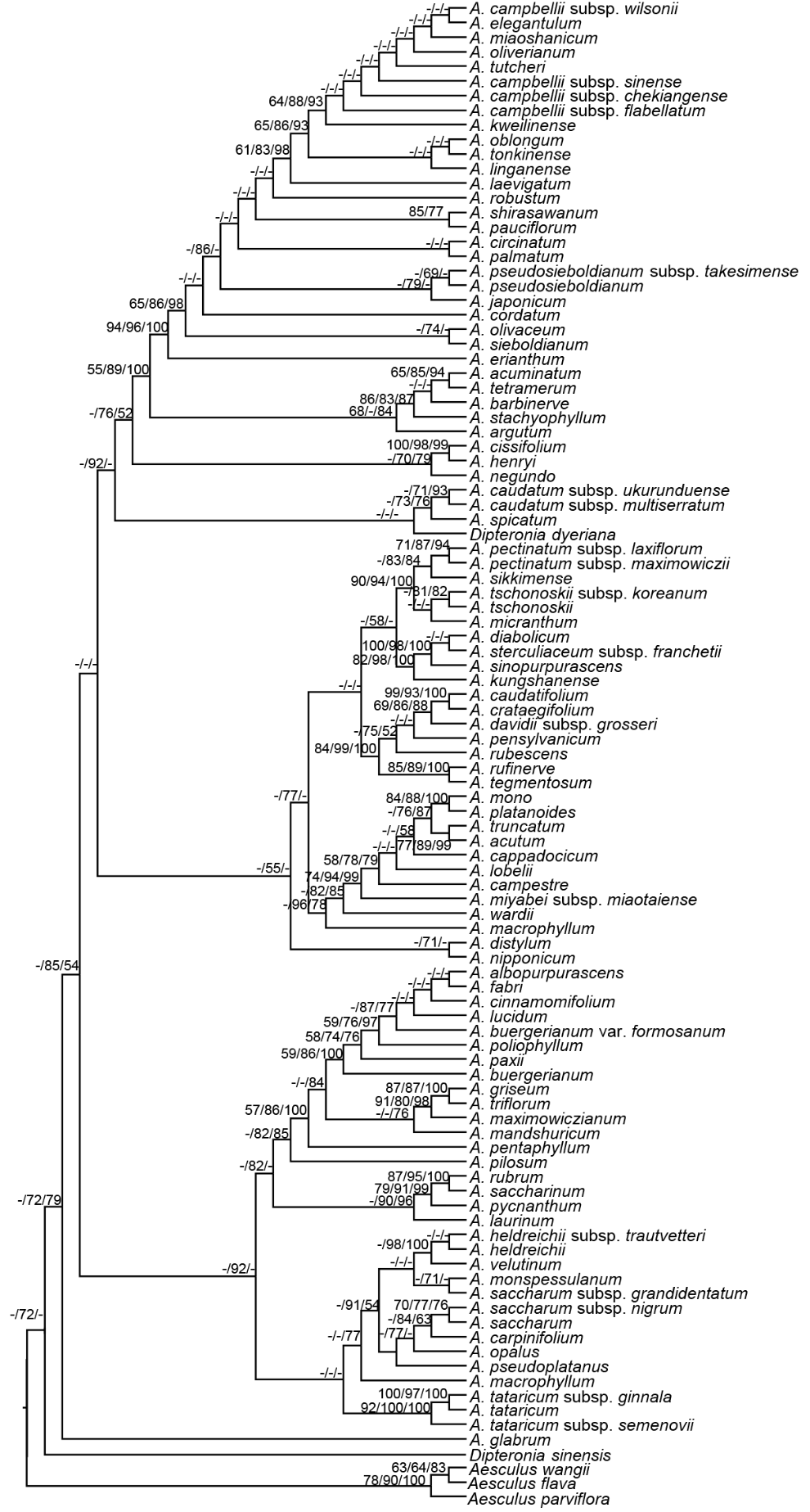


**Figure S1.** The best maximum likelihood tree of ITS region. The tree is rooted using *Aesculus* and *Dipteronia* as outgroups. Bootstrap values of MP, ML above 50% and posterior support value of BI above 0.5 are shown successively. The diagram was generated by Microsoft PowerPoint 2019 (https://www.microsoft.com/zh-cn/microsoft-365/powerpoint).


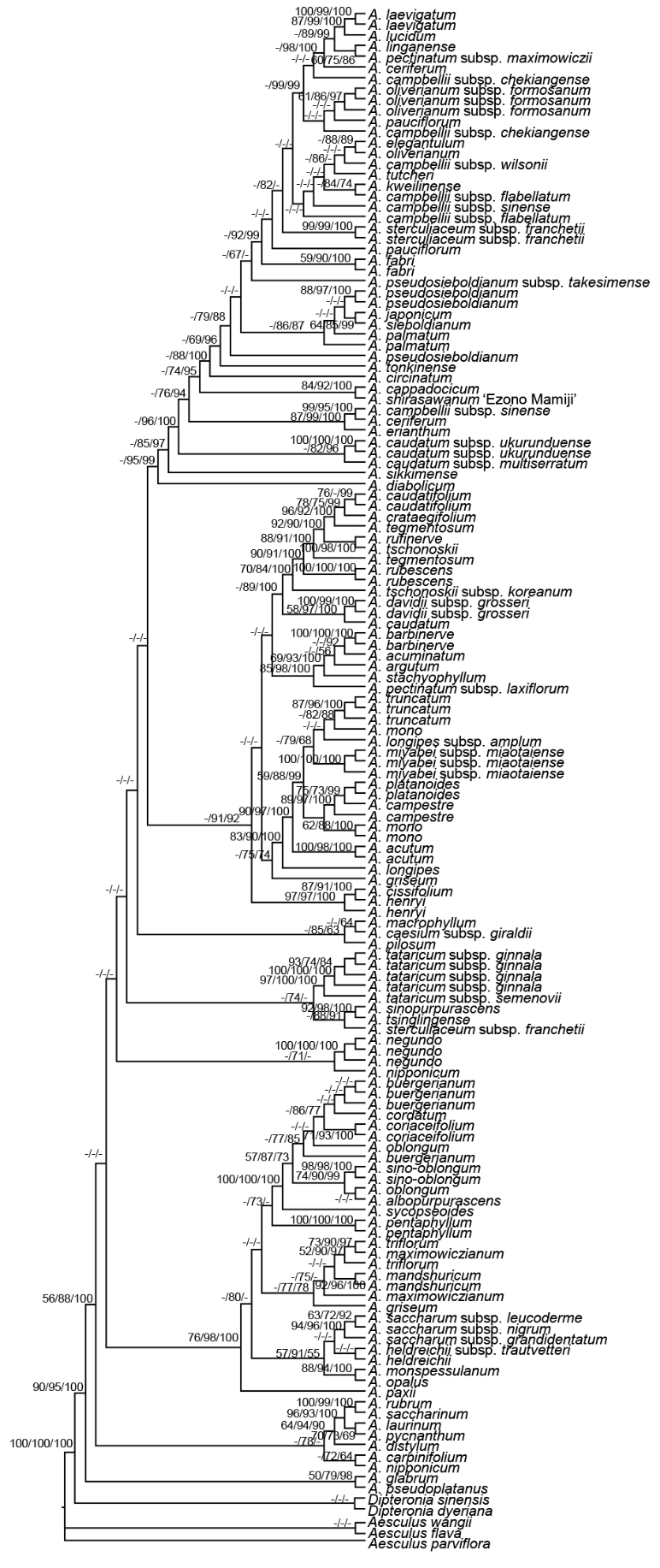


**Figure S2.** The best maximum likelihood tree combined chloroplast *rpl16*, *trnH*-*psbA* and *trnL*-*F* regions. The tree is rooted using *Aesculus* and *Dipteronia* as outgroups. Bootstrap values of MP, ML above 50% and posterior support value of BI above 0.5 are shown successively. The diagram was generated by Microsoft PowerPoint 2019 (https://www.microsoft.com/zh-cn/microsoft-365/powerpoint).


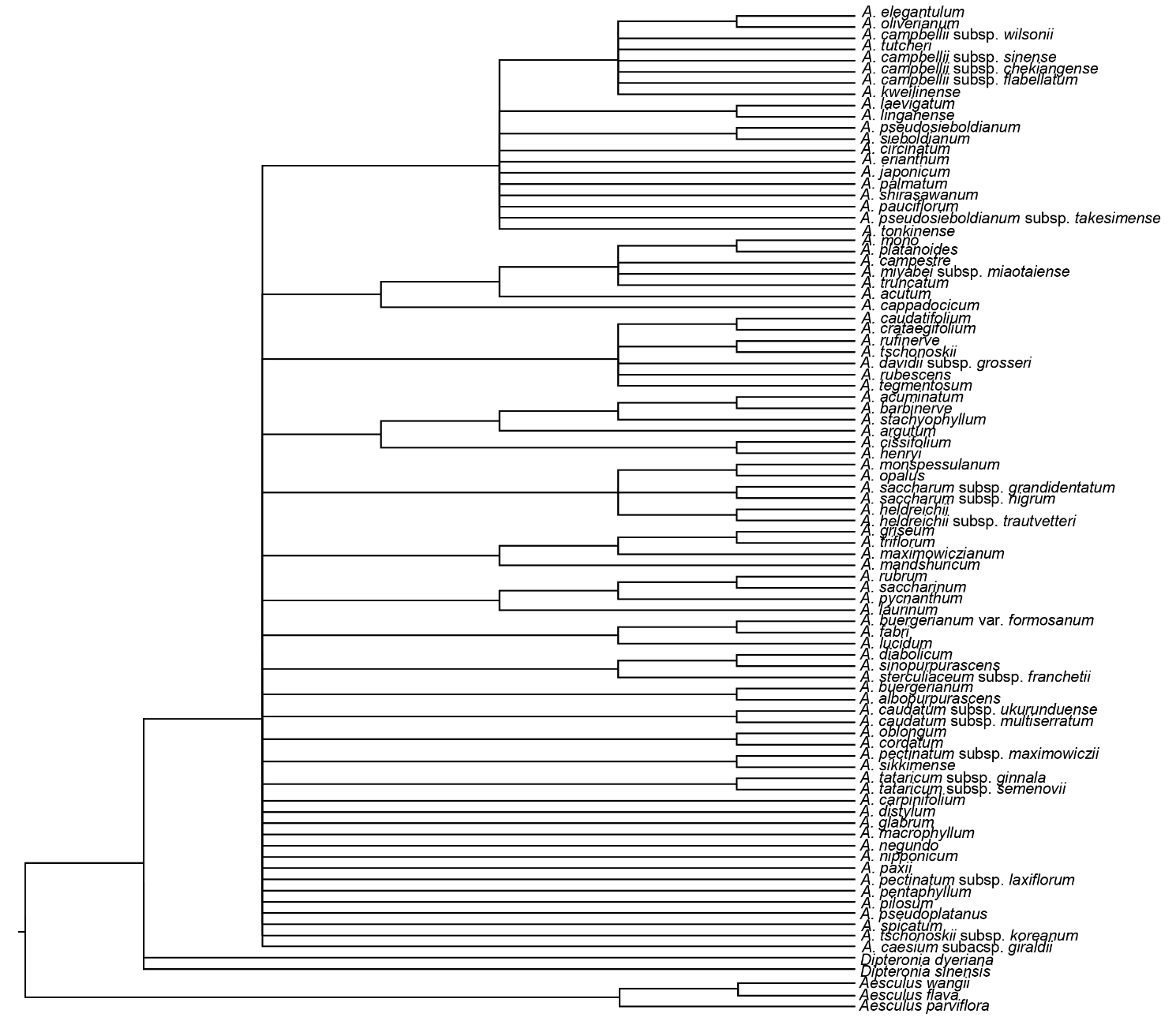


**Figure S3.** The best [maximum](http://cn.bing.com/dict/clientsearch?mkt=zh-CN&setLang=zh&form=BDVEHC&ClientVer=BDDTV3.5.0.4311&q=%E6%9C%80%E5%A4%A7%E4%BC%BC%E7%84%B6%E6%B3%95) parsimony tree combined nuclear ITS and three cpDNA fragments (*psbA*-*trnH*, *rpl16* and *trnL*-*trnF*). The tree is rooted using *Aesculus* and *Dipteronia* as outgroups. The diagram was generated by Microsoft PowerPoint 2019 (https://www.microsoft.com/zh-cn/microsoft-365/powerpoint).


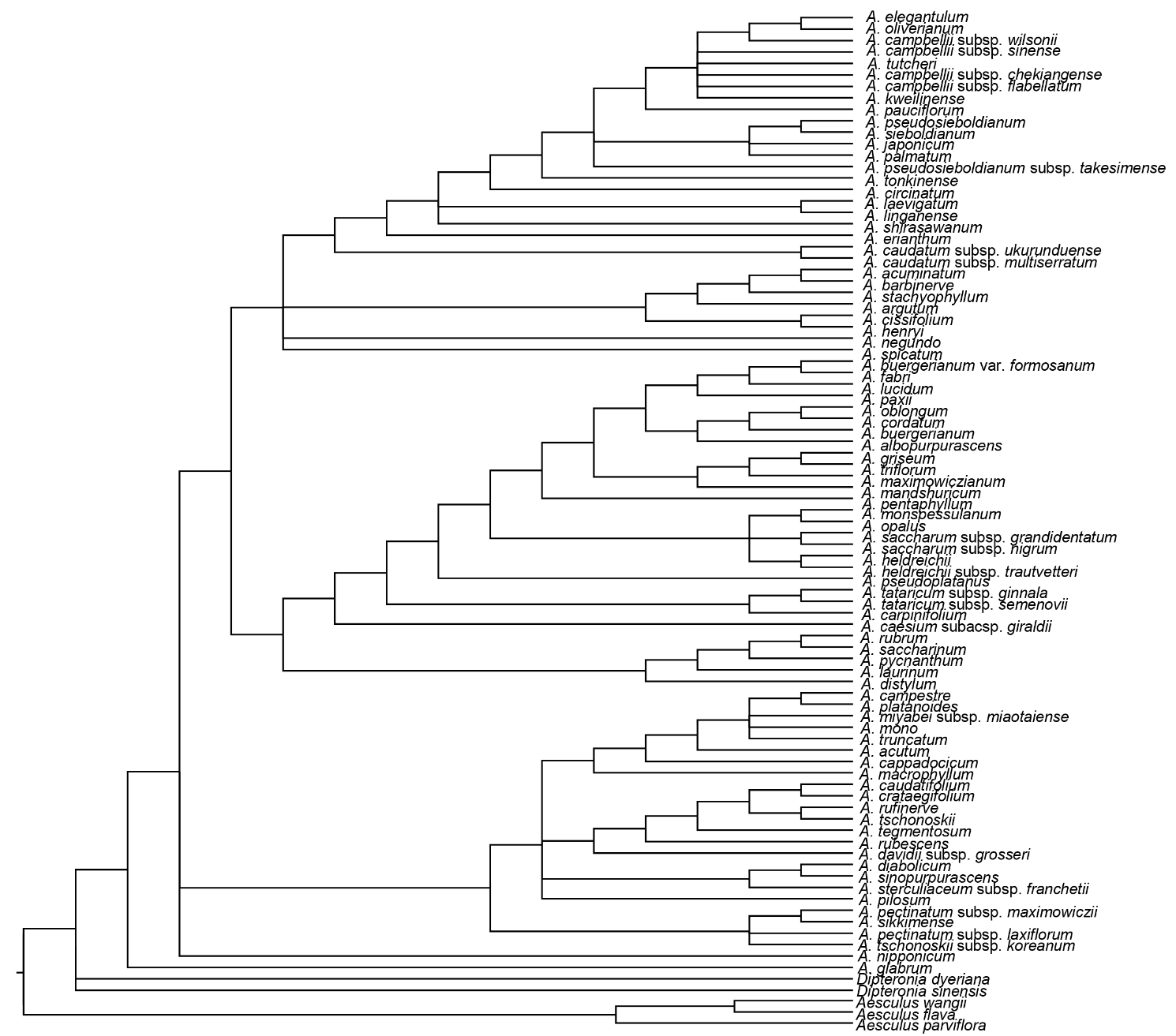


**Figure S4.** The best Bayesian analyses tree combined nuclear ITS and three cpDNA fragments (*psbA*-*trnH*, *rpl16* and *trnL*-*trnF*). The tree is rooted using *Aesculus* and *Dipteronia* as outgroups. The diagram was generated by Microsoft PowerPoint 2019 (https://www.microsoft.com/zh-cn/microsoft-365/powerpoint).

**Table S1.** Sources and GenBank accession numbers of the studied species.

| **Species** | **Native geographic region** | **Source** | **Collector** | **Genes and their GenBank accession numbers** | | | |
| --- | --- | --- | --- | --- | --- | --- | --- |
|  |  |  |  | **ITS** | ***rpl16* intron** | ***trnL*-*F* spacer** | ***psbA*-*trnH* spacer** |
| *Aesculus flava* Sol. | Central and eastern United States. | NCBI | - | MK334591 | DQ978509 | DQ978573 | DQ978642 |
| *Aesculus parviflora* Walt. | Southeastern United States. | NCBI | - | MK334616 | DQ978510 | DQ978574 | DQ978643 |
| *Aesculus wangii* Hu | Southeast Yunnan Province, China. | NCBI | - | AF406968 | AF459464 | AF411085 | KC510180 |
| *Dipteronia dyeriana* Henry | Southeast Yunnan, and southwest Guizhou Provinces, China, at 2000-2500 m. | NCBI | - | AF401120 | DQ978512 | DQ978576 | DQ978645 |
| *Dipteronia sinensis* Oliver | Southwest Henan, south Shaanxi, southeast Gansu, west Hubei, Sichuan, and Guizhou Provinces, China, at 1000-2000 m. | Qingxiushan, Nanning, China | Z-C. Lu | AF401121 | KU500460* | KU522513* | KU500519* |
| *Acer* *acuminatum* Wallich ex D. Don | Western part of the Himalayas in Nepal. Also in west Pakistan. | NCBI | - | AY605370 | DQ978449 | DQ978516 | DQ978580 |
| *A. acutum* W. P. Fang | South Anhui, South Henan, Jiangxi, and, Zhejiang Provinces, China, at 800-1100 m. | Hangzhou Botanical Garden, Hangzhou, China | J. Gao | KU902474 | KU500429* | KU522488* | KU500492* |
| *A.* *albopurpurascens* Hayata | Taiwan. | Lugu Township, Nantou, Taiwan | B-H. Huang | FN651721 | KU500440* | KU522489* | KU500493* |
| *A. argutum* Maximowicz | Mountain forests in Japan, especially on Honsu and Shikoku islands, at 600-100 m, growing along small rivers and streams. | NCBI | - | AF401153 | DQ978450 | AF401164 | DQ978581 |
| *A. barbinerve* Maximowicz | Valley of the Upper Ussuri; and Shaanxi Province, China. Also Korea. | Antu County, Yanbian, China | Q-J. Liu | AJ634569 | KU500434* | KU522490* | KU500513* |
| *A. buergerianum* Miquel | Very common in eastern China. | Qingxiushan, Nanning, China | Z-C. Lu | AF401133 | KU500435* | KU522491* | KU500514* |
| *A*. *buergerianum* Miquel var. *formosanum* (Hayata ex Koidz) Sasaki | Taiwan. | NCBI | - | FN651676 | DQ978452 | DQ978518 | DQ978583 |
| *A. caesium* subsp. *giraldii* (Pax) Murray | Southern Shaanxi, southeastern Gansu, western Hubei, Sichuan, northwestern Yunnan, and southeastern Xizang provinces, China. | NCBI | - | DQ366121 | DQ978453 | DQ978519 | DQ978584 |
| *A. campbellii* subsp*. chekiangense* (Fang) Murray | Zhejiang, northern Fujian, southern Anhui, and eastern Jiangxi Provinces, China. | Qingxiushan, Nanning, China | Z-C. Lu | KU902497 | KU500448* | KU522496* | KU500505* |
| *A. campbellii* subsp*. flabellatum* (Rehder) Murray | Hubei and Sichuan Provinces, China, at 1800-2600 m. Also occurring in Yunnan Province, northern Laos, and Vietnam. | Taian Arboretum, Taian, China | C-R. Li | KU902481 | KU500454* | KU522497* | KU500500* |
| *A. campbellii* subsp*. sinense* (Pax) de Jong, comb. & stat. nov. | Hubei and Sichuan Provinces, China, at 1500-2000 m. | South China Botanical Garden, Guangzhou, China | S-G. Jian | KU902501 | KU500451* | KU522498* | KU500510* |
| *A. campbellii* subsp*. wilsonii* (Rehder) de Jong, comb. & stat. nov. | Hubei, Yunnan, Zhejiang, Guangdong Provinces, China, at 1200-1800 m. | Qingxiushan, Nanning, China | Z-C. Lu | AF401125 | KU500450* | KU522502* | KU500511* |
| *A. campestre* L. | Widespread in Europe and western Asia; also grows in limited areas of North Africa. | Qingxiushan, Nanning, China | Z-C. Lu | AF401158 | KU500442* | KU522504* | KU500496* |
| *A. cappadocicum* Gleditsch | Northern Turkey, Caucasia, and Iran. | Kunming Botanical Garden, Kunming, China | M-J. Wang | AF401138 | KU500458* | KU522505* | KU500498* |
| *A. carpinifolium* Siebold & Zuccarini | Japan, in mountain forests up to subalpine heights. | NCBI | - | AF401148 | DQ978457 | DQ978523 | DQ978588 |
| *A. caudatifolium* Hayata | Taiwan. | Yangmingshan Park, Taiwan | B-H. Huang | DQ238380 | KU500443* | KU522507* | KU500518* |
| *A. caudatum* subsp. *multiserratum* (Maximowicz) Murray | Gansu to Yunnan Provinces, China. | NCBI | - | AY605435 | DQ978458 | DQ978524 | DQ978589 |
| *A. caudatum* subsp*. ukurunduense* (Trautvetter & Meyer) Murray | Northeast China and Japan. | Antu County, Yanbian, China | Q-J. Liu | AY605432 | KU500485* | KU522509* | KU500552* |
| *A. circinatum* Pursh | British Columbia, Canada, through Washington and Oregon, penetrating to northern California, USA; on riverbanks in the Cascade Range, Oregon, to 2000 m, often forming impenetrable thickets. | NCBI | - | AY605412 | DQ978460 | EF011741 | DQ978591 |
| *A. cissifolium* (Siebold & Zuccarini) K. Koch | Japan, in mountain forests, from southern Hokkaido to central Kyushu. | NCBI | - | AF401140 | DQ978461 | AF401165 | DQ978592 |
| *A. cordatum* Pax | Hubei, Zhejiang, and Fujian Provinces, China, in mountainous areas at 500-1200 m. | Guangxi Medicinal Botanical Garden, Nanning, China | Z-C. Lu | HM352654 | KU500439* | KU522492* | KU500515* |
| *A. crataegifolium* Siebold & Zuccarini | Central and southern Japan. | NCBI | - | AY605391 | DQ978462 | AJ4130931 | DQ978593 |
| *A. davidii* subsp*. grosseri* (Pax) de Jong, comb. & stat. nov. | Northern and central China, in the provinces of Hunan and Shaanxi, | Zhejiang [Agriculture](http://cn.bing.com/dict/clientsearch?mkt=zh-CN&setLang=zh&form=BDVEHC&ClientVer=BDDTV3.5.0.4311&q=%E5%86%9C%E6%9E%97) [and](http://cn.bing.com/dict/clientsearch?mkt=zh-CN&setLang=zh&form=BDVEHC&ClientVer=BDDTV3.5.0.4311&q=%E5%86%9C%E6%9E%97) [Forestry](http://cn.bing.com/dict/clientsearch?mkt=zh-CN&setLang=zh&form=BDVEHC&ClientVer=BDDTV3.5.0.4311&q=%E5%86%9C%E6%9E%97) University | Q-X. Chen | AY605397 | KU500459* | KU522512* | KU500550* |
| *A. diabolicum* Blume ex Koch | Mountain forests of Japan, in open, sunny situations. Not in Hokkaido and northern quarter of Houshu. | NCBI | - | AY605384 | DQ978465 | DQ978529 | DQ978596 |
| *A. distylum* Siebold & Zuccarini | Endemic to Japan, northern half of Honshu. | NCBI | - | AF401155 | DQ978466 | AF401172 | DQ978597 |
| *A. elegantulum* Fang & Chiu | Zhejiang and Jiangxi provinces, China, at 700-1000 m. | Zhejiang [Agriculture](http://cn.bing.com/dict/clientsearch?mkt=zh-CN&setLang=zh&form=BDVEHC&ClientVer=BDDTV3.5.0.4311&q=%E5%86%9C%E6%9E%97) [and](http://cn.bing.com/dict/clientsearch?mkt=zh-CN&setLang=zh&form=BDVEHC&ClientVer=BDDTV3.5.0.4311&q=%E5%86%9C%E6%9E%97) [Forestry](http://cn.bing.com/dict/clientsearch?mkt=zh-CN&setLang=zh&form=BDVEHC&ClientVer=BDDTV3.5.0.4311&q=%E5%86%9C%E6%9E%97) University | Q-X. Chen | KU902487 | KU500462* | KU522499* | KU500536* |
| *A. erianthum* Schwerin | Sichuan, Guangxi, and Hubei Provinces, China, at 2000-3000 m. | NCBI | - | AY605414 | DQ978467 | DQ978531 | DQ978598 |
| *A. fabri* Hance | Fujian, Hubei, Sichuan, Hainan, Guangdong, Guangxi, and Zhejiang provinces, China. | Chongqing, China | L-L. Zheng | AF241486 | KU500461* | KU522500* | KU500499* |
| *A. glabrum* Torrey | Rocky Mountains of North America on riverbanks and also as undergrowth in forests. | NCBI | - | AF401139 | DQ978468 | AF401181 | DQ978599 |
| *A. griseum* (Franchet) Pax | Widespread in China in the provinces of Shaanxi, Sichuan, Hubei, Henan, Guizhou, Jiangxi, Anhui, and Hunan. | Duheyuan National Nature Reserve, Shiyan, China | D-G. Zheng | AF401131 | KU500463* | KU522514* | KU500530* |
| *A. heldreichii* Orphanides ex Boissier | Northern Greece, as well as in the mountains of Albania, Yugoslavia (Serbia, Hercegovina, Montenegro), and Bulgaria. | NCBI | - | AY605304 | DQ978470 | DQ978533 | DQ978601 |
| *A. heldreichii* subsp*. trautvetteri* (Medvedev) Murray | Northeastern Turkey and the Caucasus. | NCBI | - | AM238281 | DQ978505 | DQ978569 | DQ978638 |
| *A. henryi* Pax | Central China, Hubei and Sichuan provinces. | Qingxiushan, Nanning, China | Z-C. Lu | AY605403 | KU500464* | KU522515* | KU500522* |
| *A. japonicum* Thunberg ex Murray | Mountain forests in northern Japan. | NCBI | - | AY605421 | DQ978471 | AJ413167 | DQ978602 |
| *A. kweilinense* Fang & Fang f. | Guangxi Province, China, on hills, at 1000-1500 m. | NCBI | - | KU902496 | EF186778 | EF186775 | EF186781 |
| *A. laevigatum* Wallich | Sichuan, Guizhou, Yunnan, Shaanxi, and Hubei Provinces, China; Nepal. | Shanghai Botanical Garden, Shanghai, China | B-M, Du et al. | DQ238398 | KU500465* | KU522516* | KU500523* |
| *A laurinum* Hasskarl | Upper Burma and Hainan Province, China; southeast Asia; Malaysia, Indonesia on the islands of Java and Sumatra Philippines, small Sunda islands to Flores. Grows in mountain forests at 1000-2000 m. | NCBI | - | DQ366114 | DQ978473 | DQ978536 | DQ978604 |
| *A. linganense* Fang & Chiu | Zhejiang Province, China, at 600-1300 m. | Zhejiang [Agriculture](http://cn.bing.com/dict/clientsearch?mkt=zh-CN&setLang=zh&form=BDVEHC&ClientVer=BDDTV3.5.0.4311&q=%E5%86%9C%E6%9E%97) [and](http://cn.bing.com/dict/clientsearch?mkt=zh-CN&setLang=zh&form=BDVEHC&ClientVer=BDDTV3.5.0.4311&q=%E5%86%9C%E6%9E%97) [Forestry](http://cn.bing.com/dict/clientsearch?mkt=zh-CN&setLang=zh&form=BDVEHC&ClientVer=BDDTV3.5.0.4311&q=%E5%86%9C%E6%9E%97) University | Q-X. Chen | HM352660 | KU500452* | KU522517* | KU500501* |
| *A. lucidum* Metcalf | Guangdong Province, Chian, at 800-1200 m. | Shanghai Botanical Garden, Shanghai, China | B-M, Du et al. | KU902498 | KU500467* | KU522520* | KU500504* |
| *A. macrophyllum* Pursh | Alaska to southern California in the United States, as large trees mixed in the conifer forests of the Pacific coast. | NCBI | - | AF401156 | DQ978474 | AF401160 | DQ978605 |
| *A. mandshuricum* Maximowicz | Northeast China, in mountain forests along the Upper Ussuri River; also in North and South Korea. Very abundant in the region of Vladivostok in eastern Siberia (Russia). | Antu County, Yanbian, China | Q-J. Liu | AF401129 | KU500468* | KU522521* | KU500526* |
| *A. maximowiczianum* Miquel | Japan; western Hubei and Anhui provinces, China. | Lushan Botanical Garden, Jiujiang, China | J. Gao | AJ698721 | KU500484* | KU522522* | KU500502* |
| *A. miyabei* subsp*. miaotaiense* (Tsoong) Murray | Shaanxi Province, China. | Tianmu Mountain, Linan, China | J. Gao et al. | KU902490 | KU500430* | KU522524* | KU500553* |
| *A. mono* Maximowicz | China, Mongolia, southeastern Siberia, Korea, and Sakhalin; also common in Japan on Hokkaido, Honshu, Shikoku, and Kyushu islands. | Heilongjiang Forest Botanical Garden, China | Z-J. Mao | AF241491 | KU500431* | KU522525* | KU500534* |
| *A. monspessulanum* L. | Mediterranean area: Spain, Portugal, Greece, Italy, Turkey, Southern parts of Ukraine and Georgia, southern France, occasionally in Germany in sunny and dry valleys of the rivers Mosel, Rhine, and Nahe; also in the Swiss Algeria. Grown in the milder parts of North America. | NCBI | - | AF401127 | DQ978478 | AF401161 | FN689729 |
| *A. negundo* L. | Indigenous to Canada, often forming immense impenetrable thickets; also along edges of roadsides. Found also in mixed forests in the eastern and middle regions of the United States, on riverbanks, westward to the Naturalized in eastern China. | South China Botanical Garden, Guangzhou, China | S-G. Jian | AF401142 | KU500471* | KU522527* | KU500535* |
| *A. nipponicum* Hara | Endemic to Japan, in mountain forests on Honshu and Shikoku, also on Kyushu Island, at 900-1800 m. | NCBI | - | AF401157 | DQ978480 | DQ978543 | DQ978612 |
| *A. oblongum* Wallich ex DC. | Himalayas, Nepal, Kashmir, western China, on acid soils in mountainous regions, at 600-2000 m, in a diversity of climates. | Shanghai Botanical Garden, Shanghai, China | B-M, Du et al. | AF241494 | KU500437* | KU522494* | KU500494* |
| *A. oliverianum* Pax | Hubei and Yunnan Provinces, China, in mountainous regions at 1500-2000 m. | Qingxiushan, Nanning, China | Z-C. Lu | KU902485 | KU500453* | KU522528* | KU500537* |
| *A. opalus* Miller | Jura Mountains in Switzerland and France, on hillsides up to about 1000 m altitude. Also in Burgundy in France, the Pyrenees in Spain, Corsica, the Apennines in Italy, eastward to the Caucasus, Morocco, and Algeria. | NCBI | - | AF401128 | AF459474 | DQ978545 | DQ978614 |
| *A. palmatum* Thunberg ex Murray | Widespread in Japan, Korea, Taiwan, eastern China. | Institute of Botany, Chinese Academy of Sciences, Beijing, China | P-C. Liao et al. | AF401123 | KU500472* | KU522530* | KU500503* |
| *A. pauciflorum* Fang | Zhejiang Province, China, at 200-300 m. | Hangzhou Botanical Garden, Hangzhou, China | J. Gao | HM352661 | KU500447* | KU522501* | KU500524* |
| *A. paxii* Franchet | Southwestern China, in mountain forests of Yunnan Province. | Kunming Botanical Garden, Kunming, China | M-J. Wang | AF401132 | KU500483* | KU522531* | KU500547* |
| *A. pectinatum* subsp*. laxiflorum* (Pax) Murray | China, mountains in Sichuan and Yunnan Provinces. Also on Emei shan and near Kangding. | Mao County, China | W-K Bao | HM008386 | KU500487* | KU522532* | KU500525* |
| *A. pectinatum* subsp*. maximowiczii* (Pax) Murray | Hubei, Gansu, and Sichuan Provinces, China. | Qingxiushan, Nanning, China | Z-C. Lu | HM008402 | KU500457* | KU522533* | KU500527* |
| *A. pentaphyllum* Diels | Yalung Valley, Sichuan Province, western China. | Mao County, China | W-K Bao | AF401137 | KU500474* | KU522534* | KU500538* |
| *A. pilosum* Maximowicz | Jiangxi, Shaanxi, and Gansu Provinces, China. | Institute of Botany, Chinese Academy of Sciences, Beijing, China | P-C. Liao et al. | DQ238345 | KU500491* | KU522535* | KU500546* |
| *A. platanoides* L. | Across northern Europe: in Sweden and Norway; in Finland up to 62°latitude, in Belarus, Russia, and the Baltic States as far north as Lake Ladoga and as far south as the Crimea and Caucasus but not crossing the Urals; also occurring in the central European countries, but not found in the United Kingdom nor in the coastal countries of the continent, although widely cultivated there and in North America. | Institute of Botany, Chinese Academy of Sciences, Beijing, China | P-C. Liao et al. | AF401136 | KU500475* | KU522536* | KU500540* |
| *A. pseudoplatanus* L. | Europe. | NCBI | - | AM238254 | DQ978488 | DQ978551 | DQ978620 |
| *A. pseudosieboldianum* (Pax) Komarov | Ussuri River, China; Korea. | Institute of Botany, Chinese Academy of Sciences, Beijing, China | P-C. Liao et al. | AB683974 | KU500477* | KU522539* | KU500539* |
| *A.* *pseudosieboldianum* subsp. *takesimense* (Nakai) de Jong comb. & stat. nov. | Ullenung a small Korean island. | NCBI | - | U57777 | DQ978501 | AJ413157 | HM352727 |
| *A. pycnanthum* Koch | Japan. | NCBI | - | AM113529 | DQ978490 | DQ978554 | DQ978623 |
| *A. rubescens* Hayata | Taiwan. | Xiulin Township, Hualien, Taiwan | B-H. Huang | HM008403 | KU500470* | KU522540* | KU500549* |
| *A. rubrum* L. | Eastern United States as far north and west as central Canada and the Dakotas, south to Florida, Texas, and New Mexico. | NCBI | - | AF401150 | DQ978491 | AF401182 | DQ978624 |
| *A. rufinerve* Siebold & Zuccarini | Japan, in mountain forests up to 2500 m. | NCBI | - | AY605399 | DQ978492 | AJ413094 | HM008583 |
| *A.* *saccharinum* L. | Eastern North America from Quebec to Florida, west to Minnesota, Kansas, Oklahoma. | NCBI | - | AF401151 | DQ978493 | AF401167 | DQ978626 |
| *A. saccharum* subsp. *grandidentatum* (Torrey & Gray) Desmarais | In the Rocky Mountains and Utah in the United States, on a north-south axis, at 1500-2500 m. It also grows in Texas and Oklahoma, west to Arizona, and north into Montana; extending south into northern Mexico. | NCBI | - | AM238323 | DQ978494 | DQ978558 | DQ978627 |
| *A. saccharum* subsp. *nigrum* (Michaux f.) Desmarais | Eastern and central North America; distributed form Ontario and southern Quebec southward to Kentucky, westward through Michigan to Kansas and Iowa. | NCBI | - | AY605324 | DQ978496 | DQ978560 | DQ978629 |
| *A. shirasawanum* | Japan, southern Honshu, Shikoku Kantoo district, at 1200-1800 m. | Shanghai Chenshan Botanical Garden | B-M. Du | AY605427 | KU500473* | KU522506* | KU500507* |
| *A. sieboldianum* Miquel | Japan. | NCBI | - | AF020377 | DQ978498 | DQ978562 | DQ978631 |
| *A. sikkimense* Miquel | Eastern Himalayas, Sikkim, Bhutan Assam, North Burma, in mountain forests at 2500-3000 m. Also in Yunnan Province, China. | Taian Arboretum, Taian, China | C-R. Li | HM008387 | KU500456* | KU522541* | KU500531* |
| *A. sinopurpurascens* Cheng | District of Tien-mu shan, Zhejiang Province, China. | Zijin Mountain, Nanjing, China | Z-W. Ge | KU902483 | KU500490* | KU522538* | KU500545* |
| *A. spicatum* Lamarck | In the United States and Canada, from Newfoundland to Saskatchewan, Iowa, Michigan, and Pennsylvania to northern Georgia in the Appalachians. | NCBI | - | AF401122 | DQ978499 | DQ978563 | DQ978632 |
| *A. stachyophyllum* Hiern | Himalayas, form Sikkim to Hubei Province, central China, in mountain forests to 3000 m. | NCBI | - | AY605373 | DQ987826 | DQ987824 | DQ987828 |
| *A. sterculiaceum* subsp. *franchetii* (Pax) Murray | Hubei, Yunnan, Guangxi, and Guizhou Provinces in China, and Xizang Province in Tibet, in mountainous regions at 1600-3000 m; also grows on Emei shan (Mountain Emei) in Sichuan Province, China. | Qingxiushan, Nanning, China | Z-C. Lu | DQ366145 | KU500478* | KU522543* | KU500520* |
| *A. tataricum* subsp. *ginnala* (Maximowicz) Wesmael | Northeastern and northern China, North Korea, and Japan. | Heilongjiang Forest Botanical Garden, China | Z-J. Mao | AF401147 | KU500479* | KU522544* | KU500521* |
| *A. tataricum* subsp. *semenovii* (Regel & Herder) Murray | North Afghanistan, Bokhara, Uzbekistan; Turkestan, Tien-shan, in mountain forests in the Alatan and Iliareas of Kazachstan. | Institute of Botany, Chinese Academy of Sciences, Beijing, China | P-C. Liao et al. | AY605365 | KU500480* | KU522545* | KU500543* |
| *A. tegmentosum* Maximowicz | In most lications and along the banks of the Amur and Ussuri rivers in Russia, and in mountainous regions of North Korea and Northeast China. | Changbai Mountain, China | C. Tian et al. | AF401145 | KU500481* | KU522546* | KU500551* |
| *A. tonkinense* Lecompte | Guangxi and Yunnan Provinces, China, at low altitudes; Tonkin, North Vietnam. | Shanghai Botanical Garden, Shanghai, China | B-M, Du et al. | HM352664 | KU500486* | KU522547* | KU500533* |
| *A. triflorum* Komarov | Shaanxi Province, northern China; Korea. | Institute of Botany, Chinese Academy of Sciences, Beijing, China | P-C. Liao et al. | AF401130 | KU500482* | KU522523* | KU500532* |
| *A. truncatum* Bunge | Northern China as a forest tree; also Amur, Sakhalin, Japan, Korea. | Song County, Luoyang, China | L-H. LI | AY605459 | KU500432* | KU522548* | KU500542* |
| *A. tschonoskii* Maximowicz | Northern Japan. | NCBI | - | AF020372 | DQ978508 | DQ978572 | DQ978641 |
| *A. tschonoskii* subsp*. koreanum* Murray | Mountains of North and South Korea, also in northern China. | Antu County, Yanbian, China | Q-J. Liu | DQ238385 | KU500488* | KU522549* | KU500529* |
| *A. tutcheri* Duthie | Guangdong, Guangxi and Yunnan Provinces, China, at 2500 m. | South China Botanical Garden, Guangzhou, China | S-G. Jian | KP093225 | KU500455* | KU522503* | KU500508* |

* Sequences obtained in this study.

**Table S2.** Primer pairs used for PCR amplification and sequencing for DNA fragment sequence.

| **Region** | **Primer Sequences (5′ - 3′)** | **References^a^** |
| --- | --- | --- |
| *trnL-trnF* | *trnL*: cgaaatcggtagacgctacg  *trnF*: atttgaactggtgacacgag | Taberlet et al., 1991  Taberlet et al., 1991 |
| *psbA-trnH* | *psbA*: GTTATGCATGAACGTAATGCTC  *trnH*: CGCGCATGGTGGATTCACAATCC | Sang et al., 1997  Tate et al. 2003 |
| *rpl16* | F: GCTATGCTTAGTGTGTGACTCGTTG  R: CTTCCTCTATGTTGTTTACG | Jordan et al., 1996  Asmussen 1999 |
| ITS | ITS5: GGAAGGAGAAGTCGTAACAAGG  ITS4: TCCTCCGCTTATTGATATGC | Baum et al.,1998  Baum et al.,1998 |

^a^ References:

Asmussen C B. 1999. Toward a chloroplast DNA phylogeny of the tribe Geonomeae (Palmeae). In A. Henderson, and F. Borchsenius eds. Evolution, variation, and classification of palms. Mem. New York Bot. Gard. 83: 121–129.

Jordan W C, Courtney M W, Neigel J E. 1996. Low levels of intraspecific genetic variation at a rapidly evolving chloroplast DNA locus in North American duckweeeds (Lemnaceae). Am. J. Bot. 83: 430–439.

Sang T, Crawford D J, Stuessy T F. 1997. ChloroplastDNAphylogeny, reticulate evolution, and biogeography of Paeonia (Paeoniaceae). Am. J. Bot. 84: 1120–1136.

Taberlet P L, Gielly G, Pautou J, Bouvet J. 1991. Universal primers for amplification of three non-coding regions of chloroplast DNA. Plant Mol. Biol. 17: 1105–1109.

Tate J A, Simpson B B. 2003. Paraphyly of Tarasa (Malvaceae) and diverse origins of the polyploid species. Syst. Bot. 28: 723–737.

Baum D, Mall R, Wendel J. 1998. Biogeography and floral evolution of Baobabs (*Adansonia*, Bombacaceae) as inferred from multiple data sets. Syst. Bio. 47(2): 181-207.
